# Evaluating the Stability of Open Bite Treatments and Its Predictive Factors in the Retention Phase during Permanent Dentition

**Published:** 2015-03

**Authors:** Parisa Salehi, Hamid Reza Pakshir, Seyed Ali Reza Hoseini

**Affiliations:** a Orthodontic Research Center, Dept. of Orthodontics, School of Dentistry, Shiraz University of Medical Sciences, Shiraz, Iran.; b Post graduate Specialist in Orthodontics, Member of Orthodontic Research Center, Dept. of Orthodontics, School of Dentistry, Shiraz University of Medical Sciences, Shiraz, Iran.

**Keywords:** Open bite, Relapse, Stability, Retention

## Abstract

**Statement of the Problem:**

Orthodontists often find challenges in treating the anterior open bite and maintaining the results.

**Purpose:**

This retrospective study was aimed to evaluate the stability of corrected open bite in the retention phase during permanent dentition.

**Materials and Method:**

A total number of 37 patients, including 20 males and 17 females, with the mean age of 18±2.1 years at the beginning of the treatment were studied after correction of the anterior open bite. Overbites of the patients were measured from their lateral cephalograms before (T_1_), at the end (T_2_) and at least 3 years after the end of the treatment in the presence of their fixed retainers (T_3_).The mean overbite changes and the number of patients with open bite, due to treatment relapse, at T_3_ were calculated. The relationship between the pre-treatment factors and the treatment relapse was assessed at T_1_ and T_2_. Also the effects of treatment methods, extraction and adjunctive use of removable appliances on the post-treatment relapse were evaluated.

**Results:**

The mean overbite change during the post-treatment period was -0.46±0.7 mm and six patients (16.2%) had relapse in the follow-up recall. Cephalometric Jaraback index showed statistically significant, but weak correlation with overbite changes after the treatment (*p*= 0.035; r= -0.353). No significant difference was found between the extraction and non-extraction groups (*p*= 0.117) the use and the type of the removable appliances (*p*= 0.801).

**Conclusion:**

Fixed retainers alone are insufficient for stabilizing the results of corrected open bite. The change of overbite in the retention phase could not be predicted from cephalometric measurements. Extraction and use of adjunctive removable appliance did not have any effect on the treatment relapse.

## Introduction

Orthodontists often find challenges in treating the anterior open bite and maintaining the results. Race and age are the two variables which can affect the occurrence of anterior open bite. [1] For instance, the prevalence of open bite is more in African Americans than in Caucasians or Hispanics. [1] The prevalence of open bite in different Iranian populations and various age categories has been reported to be from 1.6% [2] to 7.8%. [3] Prevalence of open bite has been shown to be 3.8% among the students aged 9-11 in downtown Shiraz in 2000. [4]

Open bite is a multifactorial, i.e. it cannot be induced by only a single factor. [5] The underlying influential causes are oral habits, undesirable growth patterns, enlarged lymphatic tissue and mouth breathing, as well as the tongue size and position. [6]

Open bite diagnosis is based on clinical examination and cephalometric analysis. A large interlabial gap is considered to be the most significant soft tissue feature of a skeletal open bite. [7] The steepness of mandibular plane is concerned as the key skeletal finding in skeletal anterior open bite. [8]

For the treatment of growing patients with anterior open bite, various appliances have been suggested such as occipital headgear, posterior bite block, vertical chin cup, [9] palatal crib, open bite bionator [10] and functional appliances. [11]

Anterior open bite is one of the most challenging malocclusion type to be retained after treatment. [12] The difficulty of maintaining the occlusion arises from the lack of control over the tongue position and movements in open bite cases. [12] In addition to the overcorrection, different retainers have been recommended for the patients such as occipital headgears, functional appliances with posterior thick bite plane [13] or palatal cribs, [14] as well as fixed retainers.

The negative esthetic features including reverse smile line and interproximal anterior spacing are usually expected when the relapse occurs in open bite patients. Concerning the mechanical difficulty involved in retaining the vertical correction, especially in the case of incisor extrusion, dentists have found it a serious challenge to maintain the long-term stability of anterior open bite treatment. Due to the complex interaction of all possible etiologic factors, ambiguity still remains on the reasons behind instability. [15]

Continuation of vertical facial growth through adolescence, long duration of the treatment, necessity of long-term cooperation of open bite patients [16] and greater rate of the relapse in this malocclusion are the issues required for comprehensive studies on the stability of open bite treatments.

Remmors *et al.* [17] evaluated 52 patients with pre-treatment open bite and observed that 27% of successfully treated patients showed opening of the bite 5 years after treatment. The study of Jonson *et al.* [18] showed negative overlap in 25.8% of their sample group at the end of post-treatment period, Lopez Gavito *et al.* [19] found treatment relapse in more than 35% of their patients in the post-retention period. Zuroff *et al.* [20] divided 64 patients into three groups according to the amount of the pre-treatment overbite and reported that all patients had positive overbite at the post retention recall. The study of de Freitas *et al.* [21] showed no relation between the overbite at the beginning of the treatment or its change during treatment and the rate of treatment relapse. Beckman *et al.* [22] mentioned significant and positive correlation between pre-treatment SN-GoGn angle and the changes of overbite after the treatment.

Previous studies had some drawbacks such as small sample size, [23-24] relatively short follow-up periods, [25] and improper definitions of open-bite. [19] Some researchers did not mention the treatment duration and the type of retainers used at the end of treatment. [17, 20] Some other studies did not discriminate between post-retention and post-treatment periods. [18] Headgear and bite plane, as accessory appliances, have considerable effects on the treatment of anterior open bite; however, to the best of our knowledge, the stability of the treatment results has not been investigated in any study. Also, there were few studies on the amount and cause of relapse of open bite treatments in Iran. Besides, there have been considerable contradictions in the results of previous studies; the relapse rate of 38.1% in one research [21] to report of no relapse in two other studies. [20, 26]

The present study was carried out to evaluate the stability of open bite treatment results in the retention phase during permanent dentition. Also, the effect of predictive factors such as pretreatment cephalometric variables and their changes during treatment was evaluated. In addition, the influence of treatment methods (extraction versus non-extraction) and simultaneous use of removable appliances on the changes of overbite in retention phase was assessed. 

## Materials and Method

Eighty patients with anterior open bite who had received treatment in Orthodontic Department of Shiraz Dental School and a private orthodontic office during 2006 to 2010 were recruited in this study. The inclusion criteria for patient selection were based on:

1. Presence of all permanent teeth up to the first molars before treatment initiation
2. Presence of pre-treatment overbite of ≤ 0 up to -3 millimeter (mm) without obvious need for surgery (such as extreme tooth show at rest, excessive display of gingiva on smile or too much facial length)
3. Skeletal nature of open bite (30>FMA>25, 60 <Jaraback index<65%)
4. Having had a course of comprehensive orthodontic treatment with or without removable appliances
5. Use of fixed retainer (bonded flexible spiral wire 4-4) at the end of treatment for at least three years
6. No frequent fracture of fixed retainers in the retention phase
7. Availability of lateral cephalograms related to pre-treatment (T_1_), post-treatment (T_2_) and at least 3 years after the end of the treatment (T_3_)
8. Preparation of lateral cephalograms of each patient with the same X-ray machine
9. No prosthodontic treatment or replacement in the incisal regions

Thirty-seven patients (20 males, 17 females) fulfilled these criteria and were included in this study. All patients were treated with full fixed appliances (edgewise technique), while a number of patients additionally had high-pull headgear or bite plane or both. The range of pre-treatment overbites was 0 to -3 mm. Twenty Two patients were treated by extraction (upper premolars or upper and lower premolars) and 15 cases did not have extraction in their treatment. Since the amount of crowding was less than 3 mm which was not sufficient for extracting premolar teeth, the main reason for extraction in the first group was open bite correction. Fixed retainers (bonded flexible spiral wire 4-4; both upper and lower arches) were placed for all the subjects. Patients were re-evaluated at least 3 years after the end of treatment in the presence of these retainers.

The major sources for gaining information were lateral cephalograms of the patients at T_1_, T_2_ and T_3_,as well as clinical inspections. For each patient, analysis of lateral cephalograms at different stages was performed by the same investigator. Magnification scales were applied on each cephalogram to control the measurement errors of linear variables caused by different magnifications of ephalograms. Intra-examiner error was assessed by measuring 10 radiographs twice a week apart. The mean differences of variables between the two time periods and their standard deviation (SD), as well as the correlation coefficient between two measurements were calculated for measurement error expression.

The mean overbite of cases was obtained from the lateral cephalograms at T_1_, T_2_ and T_3_. The mean changes of overbite between T_1_ and T_2 _and between T_2_ and T_3_ were calculated. Likewise, the number of patients with open bite and their percentage was determined at T_3._

**Figure 1:**
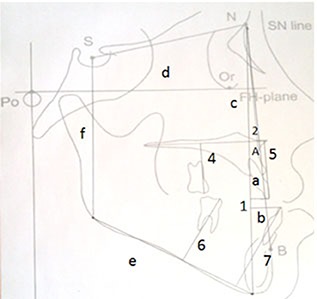
Cephalometric lines and angles used in this study

The effects of pre-treatment factors on the post-treatment relapse were studied by considering the relationship between the overbite change (T_3_-T_2_) and some factors including pre-treatment overbite, horizontal relationship of the jaws (ANB), vertical relationship of facial structures (FMA, Bjork angle and Jaraback index) and alveolar heights of molar and incisal areas of both jaws. These variables are displayed in figure 1 and represented in Table 1.

During the treatment, some factors were also evaluated that were supposed to have influence on the post-treatment relapse such as extraction versus non-extraction therapy, using or not using the removable appliances and the type of these appliances (high-pull headgear, bite plane or both). The number of patients pertaining to either one of these treatment modalities was detected and the mean changes of overbite (T_3_-T_2_) in each group was measured and compared statistically.

To enroll descriptive statistics, independent sample t-test, one-way ANOVA and Pearson and Spearman correlation coefficients were employed for quantitative data. The significance level adopted was *p*= 0.05 and SPSS Software (Statistical Package for Social Sciences) was used for analyzing the variables.

**Table 1:** Definition of cephalometric lines and angles used in the present study

**Number (in figure)**	**Variable**	**Definition**
1	Overbite	Vertical overlap of the incisal edges of maxillary (a) and mandibular incisors (b) relative to nasion-menton (N-Me) line (c)
2	ANB	The angle between the lines NA and NB
	FMA	The angle between mandibular plane (Go-Me) (e) and Frankfort line (Po-Or) (d)
	Jaraback index	The posterior facial height (S-Go) (f) relative to anterior facial height (N-Me) (c)
4	Maxillary Posterior Alveolar Height	The distance between mesiobuccal cusp of maxillary 1^st^ molar and palatal plan (ANS-PNS)
5	Maxillary Anterior Alveolar Height	The distance between incisal edge of maxillary central incisor and palatal plane
6	Mandibular Posterior Alveolar Height	The distance between mesiobuccal cusp of mandibular 1^st^ molar and mandibular plane (Go-Me)
7	Mandibular Anterior Alveolar Height	The distance between incisal edge of mandibular central incisor and mandibular plane

## Results

The Mean±SD age of samples at the beginning of the treatment was 18±2.1, the Mean±SD treatment duration was 20±3 months, and the mean follow-up period was 4 years and 2 months after treatment, with a range of 3 to 6 years.

Concerning the angles, the intra-examiner error varied from 0.23 for ANB to 0.65 for Bjork angle. While for the lines, the range of measurement errors was from 0.17 mm for mandibular posterior alveolar height to 0.76 mm for mandibular anterior alveolar height. Considering the standard deviation of measurements, the random error was small for all variables.

Table 2 displays the mean overbite of samples at T_1_, T_2_, T_3_, between T_1_ and T_2_ (T_2-_T_1_) and between T_2 _and T_3 _(T_3_-T_2_). Additionally, 16.6% (6 cases) had open bite (overbite≤0) at T_3_.

**Table 2:** Mean overbite at various stages of treatment, follow-up and mean overbite changes between the phases

	** T_1_**	** T_2_**	** T_2_-T_1_**	** T_3_**	** T_3_-T_2_**
Mean overbite (mm)	-0.63±0.76	1.62±0.50	2.25±0.62	1.16±0.88	-0.46±0.7

Pearson correlation was used to evaluate the relationship between overbite changes (T_3_-T_2_) and cephalometric variables at T_1_ (Table 3). Only Jaraback index showed statistically significant relation with post-treatment overbite changes (*p*= 0.035; r=0.353). The correlation of other variables was positive; however, not statistically significant (*p*> 0.05) (Table 3).

**Table 3:** The correlation between cephalometric measurements and post-treatment overbite change

**Cephalometric variables**	**Correlation coefficient (r)**	**P-value**
pre-treatment overbite	0.035	0.815
ANB	0.290	0.082
Bjork	0.314	0.058
FMA	0.044	0.795
Jaraback index	-0.353	0.035
Maxillary posterior alveolar height t	0.135	0.427
Mandibular posterior alveolar height	0.044	0.798
Maxillary anterior alveolar height	0.175	0.300
Mandibular anterior alveolar height	0.323	0.051

**Figure 2:**
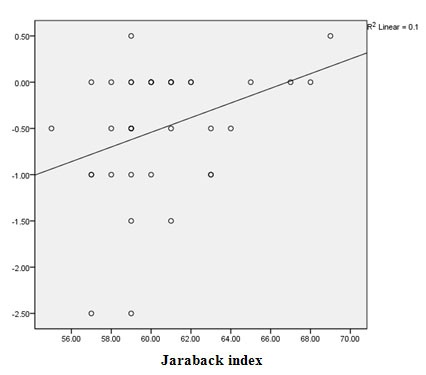
Relationship between Jaraback index and post-treatment overbite change

The relationship between Jaraback index and overbite change in the follow-up period is illustrated in figure 2. As it is shown, increase in Jaraback index lead to a decrease in the overbite changes; that is, there was a reverse correlation between Jaraback index and open bite tendency.

ANOVA test was applied for comparing the mean post-treatment overbite changes in the 3 groups with various removable appliances including (1) Headgear (HG), (2) Headgear and Bite Plane (BP) or Bite Plane and (3) No accessory appliance (Table 4). There was no significant difference among the three groups, although the mean overbite change was less in “no-appliance” group. Independent sample t-test was used to analyze and compare the means of overbite change (T_3_-T_2_) in extraction and non-extraction groups (Table 4). No significant difference was observed between the two groups; however, the mean overbite change was greater in the extraction group. Spearman correlation was used to assess the relationship between overbite changes at follow-up period (T_3_-T_2_) and changes of cephalometric measurements during the treatment (T_2_-T_1_) (Table 5). None of the radiographic variables revealed significant statistical correlation (*p*> 0.05).

**Table 4:** The relationship between the treatment modalities and mean overbite change at follow-up period

**Variable **	**Group**	**Number**	**Mean ± SD**	**Statistical index(** **F** **)**	**P-value**
Removable appliance	HG	18	-0. 52±0.83	0.224	0.801
	HG+BP Or BP	7	-0.57±0.73		
Type of treatment	No appliance	12	-0.37±0.48	0.224	0.801
	extraction	22	-0.63±0.77		
	Non extraction	15	-0.26±0.63		

**Table 5:** The relationship between changes of cephalometric variables during the treatment and post-treatment overbite changes

**Change of Cephalometric variables (** ** T_2_-T_1_) **	**Correlation coefficient(r)**	**P-value**
ANB	0.108	0.523
Bjork	0.230	0.171
FMA	0.191	0.257
Jaraback index	0.105	0.543
Maxillary posterior alveolar height	0.158	0.351
Mandibular posterior alveolar height	0.296	0.075
Maxillary anterior alveolar height	-0.069	0.687
Mandibular anterior alveolar height	0.062-	0.717

## Discussion

Based on the findings of this study, 6 patients (16/6%) experienced relapse of open bite in the presence of their fixed retainers. No relationship was found between the cephalometric variables or their changes during the treatment and the post-treatment bite opening in the studied sample. Moreover, various treatment strategies or simultaneous use of removable appliances did not show any significant difference regarding treatment stability.

Based on the results of this study, the mean overbite change during the follow-up period was found to be -0.46±0.7 mm. However, the number of patients with relapse (six open bite cases comprising 16.6%) during the post-treatment observations seems more important from clinical point of view. Remmors *et al.* [17] and Janson *et al.* [18] reported similar results in their studies. Remmors *et al.* evaluated 52 patients with pre-treatment open bite and observed that 27% of successfully treated patients showed opening of the bite 5 years after treatment. The study by Jonson *et al.* showed negative overlap in 25.8% of the samples at the end of post-treatment period; although they applied a different definition for overbite measurement. In a meta-analysis study, Greenlee *et al.* [27] reported the stability of nonsurgical treatments of anterior open bite to be greater than 75% at 12 or more months after the treatment. Lopez Gavito *et al.* [19] found treatment relapse in more than 35% of their patients in post-retention period. However, the limitation of their study was the inaccurate definition of open bite, which could likely not only be observed in deep bite patients, but also was affected by antero-posterior position of the incisors. Zuroff *et al.* [20] divided 64 patients into three groups based on the amounts of pre-treatment overbite and reported that all the patients had positive overbite at the post retention recall. However, as the authors expressed, their finding should be interpreted with caution since only 15 patients with pre-treatment open bite were present in that study.

In our study, neither the pre-treatment cephalometric measurements (primary overbite, horizontal jaw relationships, vertical relationships of facial structures and alveolar heights in the different parts of the jaws), nor the change of radiographic variables during the treatment had the capability to predict stability of the treatment. Only Jaraback index value in the pre-treatment phase showed significant- but weak- relationship with post-treatment overbite changes. As expected, a negative correlation was noticed because the increase in posterior facial height relative to the anterior part of face decreases the tendency for creating anterior open bite. Regarding this poor relationship and the possibility of chance in that, it could be possibly concluded that there was no reliable factor for predicting post-treatment changes of open bite therapies. Moreover, no significant correlation was found between the changes of Jaraback index during the treatment and the treatment stability. Considering the limitations of our study, such as retrospective design, sample size and lack of control on all variables, this conclusion should be interpreted with caution. Similarly, Lopez Gavito *et al.* [19] have not reported any relation between primary overbite, steepness of mandibular plane or any exclusive factor and post-treatment stability. The study of de Freitas *et al.* [21] also showed no relation between overbite at the beginning of the treatment or its changes during the treatment and the rate of treatment relapse. Among many factors investigated by Remmers *et al.* [17] only mandibular and palatal plane angles at the beginning of the treatment showed significant relationship with post-treatment overbite changes. Nonetheless, the researchers explained this was achieved only by chance, hence, open bite could not be predicted successfully from pre-treatment cephalometric variables. Beckman *et al.* [22] mentioned significant and positive correlation between pre-treatment SN-GoGn angle and the changes of overbite after treatment. However, inconsistent treatment techniques, broad range of participants’ age, various types of retainers and the limited number of cases at the follow-up recall influenced the results of the study.

Based on the results of our study, the extraction and non-extraction groups did not show any significant difference in terms of treatment stability. Remmers *et al.* [17] reported that extraction therapy (either in the upper or both arches) was not associated with the closure or stability of anterior open bite. Janson *et al.* [18] observed that the relapse rate of open bite treatment in extraction and non-extraction groups were 25.8% and 38.1%, respectively, with extraction therapy exhibiting greater stability. Nonetheless, neither group showed a statistically significant difference in the number of patients with open bite in the follow-up period.

The influence of using removable appliances in addition to comprehensive treatment of anterior open bite was not evaluated in the previous studies. Our investigation showed that simultaneous use of headgear or bite plane or their combination would not lead to a better stability of the treatment. It must be pointed out, however, that more accurate studies with greater control of other variables are required to assess the advantages or disadvantages of using removable appliances as adjunct to fixed appliance therapy.

Some adjunctive treatments like orofacial myofunctional therapy (OMT) [28] and non-surgical treatments with temporary anchorage devices [29] have presented promising results in recent studies; although their long-term effectiveness has not been evaluated thoroughly. [30]

Considering the relapse of treated open bite in six patients during the retention phase, it seems that fixed retainers alone were not absolutely sufficient for maintaining the results of open bite treatment. However, since this study, similar to the previous investigations, is a retrospective study, some attempts such as gaining strict control over the variables, making comparison with control groups, evaluating the impact of growth, and post-treatment factors like use of other types of retainers, patients cooperation and lengthening the follow-up period, as well as designing prospective studies are suggested to be considered for future investigations. 

## Conclusion

1. Fixed retainers were not thoroughly successful in maintaining the overlap of incisors achieved during treatment of open bite patients.
2. There was no possibility of predicting treatment stability due to the absence of correlation between pre-treatment overbite and cephalometric variables or the change of radiographic measurements during treatment.
3. No significant difference was observed between the extraction and non-extraction groups with regard to the post treatment overbite changes.
4. Simultaneous use of removable appliances and their type did not show significant differences in the rate of treatment relapse.

In addition to fixed retainers, other types of retainers for controlling interarch discrepancy and vertical dimension are suggested in the retention phase of open bite patients. 
